# The schedule of administration of canakinumab in cryopyrin associated periodic syndrome is driven by the phenotype severity rather than the age

**DOI:** 10.1186/ar4184

**Published:** 2013-02-26

**Authors:** Roberta Caorsi, Loredana Lepore, Francesco Zulian, Maria Alessio, Achille Stabile, Antonella Insalaco, Martina Finetti, Antonella Battagliese, Giorgia Martini, Chiara Bibalo, Alberto Martini, Marco Gattorno

**Affiliations:** 1 UO Pediatria II, G. Gaslini Institute and Department of Pediatrics, University of Genoa, Via Gaslini 5, Genova, 16147, Italy; 2 Department of Pediatrics, IRCCS Burlo Garofalo, University of Trieste, Via dell'Istria 65, Trieste, 34137, Italy; 3 Dipartimento A.I. di Pediatria, University of Padua, Via Giustiniani 3, Padova, 35128, Italy; 4 Department of Pediatrics, Federico II Hospital, Via Sergio Pansini 5, Napoli, 80131, Italy; 5 Department of Paediatrics, A. Gemelli Hospital, University of Rome, Largo Agostino Gemelli 8, Roma, 00168, Italy; 6 Department of Pediatrics, Ospedale Pediatrico Bambin Gesù, Piazza Sant'Onofrio 4, Roma, 00165, Italy

## Abstract

**Introduction:**

Interleukin-1 (IL-1) blockade is the treatment of choice of cryopyrin associated periodic syndromes (CAPS). Anti-IL-1 monoclonal antibody (canakinumab) was recently registered. However no clear data are available on the optimal schedule of administration of this drug. The aim of the present study was to analyse the impact of canakinumab on CAPS patients in daily clinical practice and to identify the best schedule of administration according to age and phenotype.

**Methods:**

13 CAPS patients (10 children and 3 young adults) treated with canakinumab were followed for 12 months. Clinical and laboratory parameters were collected at each visit. Health-related quality of life (HRQoL) was recorded at month 12. Complete response was defined as absence of clinical manifestations and normal examinations. Clinical and laboratory variables at last follow-up were compared with those registered at the moment of anakinra discontinuation.

**Results:**

seven patients with chronic infantile neurological cutaneous articular (CINCA) syndrome, four patients with Muckle-Wells syndrome (MWS) and two patients with an overlapping MWS/CINCA phenotype were analysed. CINCA patients experienced a higher number of modifications of the treatment (increased dosage or decreased dosing interval) in respect to MWS patients. At the end of the follow-up CINCA patients displayed a higher frequency of administration with a median dose of 3.7 mg/kg (2.1 mg/kg for MWS patients). Canakinumab was withdrawn in a patient with CINCA for incomplete response and poor compliance. The effect of canakinumab on HRQoL was similar to that observed during treatment with anakinra, with the exception of an improvement of the psychosocial concepts after the introduction of canakinumab.

**Conclusions:**

The use of canakinumab in daily practice is associated with persistent satisfactory control of disease activity but needs progressive dose adjustments in more severe patients. The clinical phenotype, rather than the age, represents the main variable able to determine the need of more frequent administrations of the drug at higher dosage.

## Introduction

Familial cold autoinflammatory syndrome (FCAS), Muckle-Wells syndrome (MWS) and chronic infantile neurological cutaneous and articular syndrome (CINCA) represent the clinical spectrum associated to mutations in *NLRP3 *gene coding for the cryopyrin protein [1,2] and are collectively known as cryopyrin-associated periodic syndrome (CAPS).

FCAS is characterized by urticarial rash, arthralgia and fever spikes of short duration induced by cold exposure [3]. In MWS recurrent episodes of urticaria-like eruptions, fever, chills, malaise and limb pain occur from childhood onwards and are associated with the late development of sensorineural hearing loss and amyloidosis [4]. CINCA (or neonatal onset multi-systemic inflammatory disease, NOMID) represents the most severe condition and is characterized by a neonatal onset urticarial-like rash, fever, central nervous system (CNS) involvement (mental retardation, chronic aseptic meningitis, increased intracranial pressure, cerebral atrophy, ventriculomegaly, sensorineural hearing loss and chronic papilledema), chronic inflammatory arthropathy, skeletal dysplasia and specific facial and dysmorphic features [5].

Cryopyrin is involved in the assembly of an intracellular multi-protein complex (called inflammasome) that plays a pivotal role in the induction and secretion of the biologically active 17 kD form of IL-1β [6,7]. Anti-IL-1 blockers are highly effective in CAPS. The short- [8-10] and long-term [11-13] effectiveness of the IL-1 receptor antagonist (anakinra) in CAPS have been already described in the last few years. Other IL-1 inhibitors, such as rilonacept, a human dimeric fusion protein that incorporates the extra-cellular domain of both IL-1 receptor type I and IL-1 receptor accessory protein [14], and a fully human anti-IL-1β monoclonal antibody, canakinumab are also available [15].

In a recent trial the use of subcutaneous doses of 150 mg (or 2 mg/kg) of canakinumab every 8 weeks for 24 weeks was generally associated with complete control of clinical manifestations and laboratory parameters in patients with a prevalent MWS phenotype [15]. These positive results were confirmed in the following 24-month phase III trial [16]. Interestingly, in this latter study a relevant percentage of patients required modification of the treatment schedule by means of increased dosage and/or frequency of administration [16]. This was mainly observed in pediatric and CINCA patients who were not included in the previous trial. However, the description of the pattern of disease activity and the strategy used for the modified treatment schedule were not reported [16].

In this retrospective multicenter study we describe one year of follow-up in a cohort of pediatric and CAPS patients treated with canakinumab. The main aims were to 1) verify the efficacy and safety of the drug in everyday clinical practice, 2) evaluate the impact of the drug on the quality of life, and 3) identify the best schedule for CAPS patients according to their age and phenotype.

## Materials and methods

Thirteen unrelated CAPS patients (female:male ratio 7:6; 10 children, 3 adults; mean age 14.6 years, range 8.7 to 38 years) were enrolled in the study from five pediatric rheumatology centers. Twelve of these patients were previously enrolled in the CACZ885D2306 trial [16]. According to their phenotype, seven patients were classified with CINCA for the presence of early-onset urticarial skin rash associated with involvement of the CNS (papilledema, early-onset hearing loss, brain atrophy at MRI, severe headache) and bone dysplasia [2,17]. Four patients were classified with MWS syndrome (absence of CNS involvement and bone dysplasia). Two patients were defined as having an overlapping phenotype between MWS and CINCA for the presence of early-onset hearing loss and/or papilledema in the absence of brain atrophy, mental retardation or bone dysplasia. Ten patients carried mutations in the *NLRP3 *gene (Table 1).

**Table 1:** Demographic data, duration of treatment, schedule (dose and frequency) of canakinumab and response to treatment at the beginning of the follow-up period

Patient number	Phenotype	*NLRP3* mutation	Disease onset	Duration of treatment (months)	Body weight (Kg)	Dose (mg/kg)	Dose at each Administration, mg	Canakinumab frequency, weeks	Response
1	CINCA	N477K	birth	5	65	4.6	300	8	partial
2	CINCA	F573S	birth	5	38	4.0	152	8	inadequate
3	CINCA	Negative	2 months	5	50	6.0	300	8	partial
4	CINCA	Negative	birth	6	54	2.8	150	8	partial
5	MWS/CINCA	D303N/V198M	1 week	12	29	2.0	72	8	complete
6	MWS/CINCA	T348M	6 months	11	85	3.5	300	8	partial
7	CINCA	E304K	birth	12	78	3.8	300	8	partial
8	MWS	T348M	birth	12	63	2.4	150	8	partial
9	MWS	E525K	15 months	17	62	2.4	150	8	complete
10	MWS	D303N	3 weeks	20	37	2.0	74	8	complete
11	CINCA	T348M	birth	15	23	2.0	46	8	partial
12	CINCA	Negative	birth	7	61	2.5	150	8	complete
13	MWS	V198M	9 years	-	38	2.0	76	8	complete

CINCA: chronic infantile neurological cutaneous articular syndrome; MWS: Muckle-Wells syndrome; MWS/CINCA: overlap syndrome.

At the end of the CACZ885D2306 study (July 2010), all patients continued treatment with canakinumab in an open fashion with the last schedule (dose and frequency) used during the trial. Before the registration of the drug in Italy (November 2010), patients received the treatment for compassionate use, provided by Novartis, Italy. An additional patient was treated soon after the registration of the drug. The study was approved by the Ethical Board of the G. Gaslini Institute. Local ethical boards also approved the study in each center. Informed consent was obtained from patients or families. Patients were followed at their center with monthly or bi-monthly visits. According to normal daily clinical practice used in Italian centers for patients with CAPS the following parameters were recorded: 1) presence of disease-associated manifestations (skin rash, arthralgia/arthritis, myalgia, headache and conjunctivitis) and the global evaluation of disease activity with a four-point scale as absent (0), minimal (1), moderate (2), severe (3) [16]; 2) laboratory parameters, including hemogram, C-reactive protein (CRP), serum amyloid A protein (SAA); 3) eye examination and audiogram (with a frequency of 3 to 6 months according the policy at each center), and 4) a national language version of the parent-administered 50-item version of the Child Health Questionnaire (CHQ-PF 50) at month 12, as previously described [11].

Modifications of dose and/or frequency of administration of canakinumab were performed according to the judgment of the physician in charge based on the clinical picture and/or laboratory parameters. Adverse events were also registered. After 12 months of treatment, data from clinical charts were retrospectively evaluated. Response to treatment was evaluated as follows: i) complete response: absent or minimal disease activity at the global assessment with acute phase reactants within the normal range, ii) partial response: absent or minimal global disease activity associated with elevation of acute phase reactants, or iii) inadequate response: moderate or severe global disease activity associated with elevated acute phase reactants.

### Intra-individual comparison between anakinra and canakinumab

Twelve out of thirteen patients were previously treated with anakinra for a median period of 42 months (range 12 to 60 months) [11]. For these patients, clinical and laboratory variables at the time of anakinra withdrawal were retrospectively evaluated. The CHQ-PF 50 administered after 12 months of treatment with canakinumab was compared with the same evaluation performed in the same patients during anakinra treatment [11], using the non-parametric Wilcoxon pairs test.

## Results

### Baseline

At the end of the ACZ885D2306 trial seven patients (three with MWS, one with MWS/CINCA and three with CINCA) were treated at the dose of 2 mg/kg (or 150 mg if body weight was > 40 Kg) every 8 weeks, which was the starting dose used in the trial [16]. Due to incomplete control of disease activity, five patients (one with MWS/CINCA and four with CINCA) experienced increasing dosing during the trial and were therefore receiving 4 mg/kg (or 300 mg, if body weight was > 40 Kg) every 8 weeks (Table 1). The MWS patient (number 13) who was treated after the registration of the drug received the starting dose of 2 mg/kg every 8 weeks according to the terms of the marketing authorization.

At baseline four patients with the MWS or MWS/CINCA phenotype (patients number 5, 9, 10 and 13) were in complete remission, while two of them (patients 6 and 8) were in partial remission (Table 1). Among patients with CINCA, a complete or partial response was observed in one (patient 12) and five (patients 1, 3, 4, 7 and 11), respectively (Table 1). One patient (number 2) was considered a non-responder due to the presence of both clinical manifestations and elevation of acute phase reactants.

### Follow-up period

**Figure 1:**
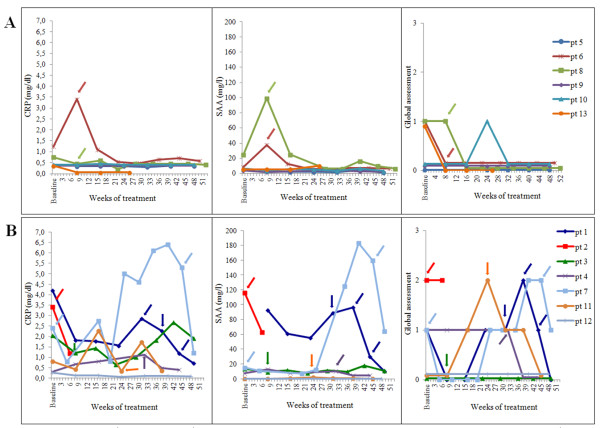
**C-reactive (CRP) in mg/dl, serum amyloid A (SAA) in mg/l and global physician assessment during 12 months of follow-up in patients with Muckle-Wells Syndrome (MWS) and overlapping MWS/chronic infantile neurological cutaneous and articular syndrome (CINCA)**. Panel A: patients with MWS and overlapping MWS/CINCA; panel B: patients with CINCA. Arrows represent changes in the treatment schedule (dose or frequency). Pt, patient.

In Figure 1 the clinical and laboratory variables during the 12 months of follow-up in patients with less severe (MWS and MWS/CINCA, panel A) and more severe (CINCA, panel B) phenotypes are shown. Of the six patients with the MWS and MWS/CINCA phenotypes, three (patients 5, 9, and 10) did not require any adjustment of the therapy due to a persistent optimal control of both clinical and laboratory parameters during the whole follow-up period (Figure 1A). Two others (patients 6 and 8) required at least one modification of the treatment schedule due to a persistent elevation of acute phase reactants (Figure 1A). After three doses, due to the persistent control of clinical and laboratory parameters, the dosing frequency for patient number 13 was decreased to every 10 weeks. At the following visit, in the light of persistent and complete wellbeing, both the physician and the parents of the patient agreed to discontinue the treatment with the aim of using the drug only on demand. At the end of follow-up the majority of MWS and MWS/CINCA patients were treated with a dose below 2.5 mg/kg. Only patient number 6, presenting a MWS/CINCA phenotype with a T348M mutation, required a higher dose (3.7 mg/kg) due to persistent elevation of acute phase reactants (Table 2).

**Table 2:** Dose of anti-IL drug, acute phase reactants, physician assessment of disease and response to treatment at last follow-up on treatment with Canakinumab and at the moment of anakinra withdrawal

	Last follow-up on canakinumab					Last follow-up on anakinra				
	
Patient number	Dose, mg (mg/Kg)/frequency	CRP, mg/dl	SAA, mg/l	Clinical assessment	Response	Dose. mg (mg/kg)/frequency	CRP, mg/dl	SAA mg/l	Clinical assessment	Response
1^1^	300 (4.30)/5 wks	1.17	29.9	mild	partial	75 (1.30)/day	0.61	25.0	absent	partial
2^1^	150 (4.00)/6 wks	1.18	ND	moderate	inadequate	75 (2.00)/day	neg	18.0	absent	partial
3^1^	300 (5.90)/6 wks	2.67	18.1	absent	partial	100 (1.80)/day	0.47	7.50	absent	partial
4^1^	150 (2.80)/6 wks	neg	neg	absent	complete	100 (2.00)/day	neg	neg	minimal	complete
5^2^	78 (2.00)/8 wks	neg	neg	absent	complete	55 (2.00)/day	neg	neg	minimal	complete
6^2^	300 (3.75)/7 wks	0.57	6.5	absent	partial	100 (1.16)/day	0.63	neg	mild	partial
7^1^	300 (3.70)/4 wks	1.20	64.0	mild	partial	100 (1.30)/day	0.73	neg	absent	partial
8^2^	150 (2.30)/6 wks	neg	neg	absent	complete	ND	ND	ND	ND	ND
9^2^	150 (2.40)/8 wks	neg	neg	absent	complete	50 (1.00)/day	neg	neg	absent	complete
10^2^	100 (2.00)/8 wks	neg	neg	absent	complete	20 (0.66)/day	neg	neg	absent	complete
11^1^	60 (2.00)/7 wks	neg	neg	absent	complete	23 (1.00)/day	neg	13.2	mild	partial
12^1^	150 (2.00)/8 wks	neg	neg	absent	complete	55 (1.00)/day	neg	neg	absent	complete
13^2^	78 (2.00)/10 wks	neg	neg	absent	complete	38 (1.00)/day	neg	neg	absent	complete

^1^Patients with the chronic infantile neurological cutaneous and articular syndrome (CINCA) phenotype; ^2^patients with the Muckle-Wells syndrome (MWS) and MWS/CINCA phenotype; CRP, C reactive protein; SAA, serum amyloid A; neg, negative; ND, not done.

Six out of seven patients with CINCA received at least one modification of the canakinumab schedule. Due to a persistent elevation of acute phase reactants associated with mild clinical manifestations, patients 3, 4 and 11 required a single modification of the frequency. In patients 1 and 7 the frequency was increased three and two times to a final schedule of 300 mg every 5 and 4 weeks, respectively (Table 2). Patient 2 was treated at baseline with 300 mg every 8 weeks. The frequency of administration of canakinumab was initially increased to every 6 weeks because of the persistence of disease activity (Figure 1B). At the following evaluation both the physician in charge and parents preferred to discontinue canakinumab due to the persistence of disease-associated symptoms (arthralgia and malaise), the presence of possible side effects (dizziness) and the remarkable elevation of acute phase reactants (Figure 1B). The patient was subsequently treated with anakinra, (2 mg/kg/day) with a complete control of the clinical manifestations and normalization of acute phase reactants.

The comparison between the two subgroups (MWS and MWS/CINCA vs CINCA) did not reveal any difference in their median age (Table 3). Conversely, during the course of the study CINCA patients experienced a higher number of adjustments (increased dose or decreased dosing interval) in respect to MWS patients (Table 3). At the end of the follow-up CINCA patients had required a higher frequency of administration and a trend towards the need for a higher median dose (3.7 vs 2.1 mg/kg) (Table 3).

**Table 3:** Comparison of age, duration of disease, final dose, frequency of administration of canakinumab and number of adjustments (dose and/or frequency) performed during 12 months in patients with MWS and CINCA

	Muckle-Wells (6 patients)^1^	CINCA (7 patients)	*P*-value^2^
Age, years, median (range)	13.6 (10.7, 23.7)	15 (8.7, 38.0)	0.56*
Disease duration, median, years (range)	13,6 (3.0, 23.2)	15 (8.7, 38.0)	0.61*
Dose, mg/kg, median (range)	2.15 (2.0, 3.7)	3.7 (2.0, 5.9)	0.16*
Frequency, weeks, median (range)	8 (6, 10)	6 (4, 8)	0.03
Adjustments/year, number	2	9	0.05

^1^Includes two patients with a Muckle-Wells syndrome (MWS)/chronic infantile neurological cutaneous and articular syndrome (CINCA) phenotype; ^2^Mann-Whitney *U*-test, *not significant.

During the 12 months of follow-up repeated audiograms and eye examinations did not show substantial changes from baseline. Flares of headache, or modifications in behavior or mental performance were not observed. Compliance with treatment was good, and no reactions at the site of the injection were recorded. Patient number 8 presented with cellulitis of the leg at month 11, which required hospitalization for intravenous antibiotic treatment. Methicillin-resistant staphylococcus aureus was isolated. The administration of canakinumab was postponed until the complete resolution of the infection, which occurred without complications.

**Figure 2:**
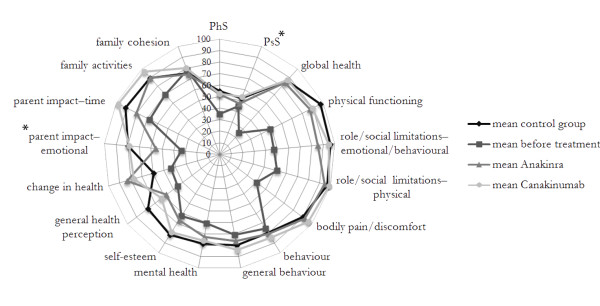
**Impact of different IL-1 blockers on health-related quality of life in patients with cryopyrin associated periodic syndromes (CAPS) evaluated by the Child Health Questionnaire (CHQ-PF 50)**. Squared boxes represent the mean values obtained from the patients before any treatment with IL-1 blockers (see also [11]). Triangles and circle boxes represent the mean values at the last follow-up with anakinra and canakinumab treatment, respectively. *P < 0.05 (Wilcoxon pairs test). PhS: physical summary score. PsS: psychosocial summary score.

The 15 subscales of the CHQ and the two summary scores, the physical summary score (PhS) and the psychosocial summary score (PsS), recorded at the time of the last follow-up and the reference values for healthy controls are reported in Figure 2. Overall, the health-related quality of life (HRQoL) of canakinumab-treated CAPS patients was generally comparable to that recorded in the control group. Patients with CAPS still presented a significantly impaired perception of general health (*P *< 0.001) and physical functioning (*P *= 0.02) when compared with healthy controls.

### Within-patient comparison of anakinra and canakinumab

Twelve out the thirteen patients treated with canakinumab were previously treated with anakinra. We therefore evaluated for each patient whether the pattern of response to canakinumab was different from that registered at the time of anakinra withdrawal. At the time of the last administration of anakinra six patients (two with CINCA, one with MWS/CINCA and three with MWS) displayed a complete response (Table 2). Six patients (five with CINCA, one with MWS/CINCA) displayed a partial response due to a slight increase of acute phase reactants (Table 2). All five patients presenting a partial response to canakinumab already showed a partial or inadequate response to anakinra (Table 2).

We also had the possibility of comparing HRQoL at the last follow-up of treatment with canakinumab to that obtained at the time of anakinra withdrawal (Figure 2) [11]. The comparison did not reveal a significant difference of the impact of the two drugs on physical concepts. Conversely, canakinumab treatment was associated with a significant amelioration of psychosocial concepts (*P *= 0.03), especially for the parental emotional perception of the disease (*P *= 0.045) with a trend towards significance for self-esteem (*P *= 0.09) and parental time (*P *= 0.1) (not shown).

## Discussion

In the present paper we describe the impact of canakinumab in the daily clinical practice in a cohort of CAPS patients mostly characterized by pediatric age and/or a severe phenotype. After one year of follow-up, twelve out of thirteen patients displayed complete control of clinical manifestations, with a slight elevation of acute phase reactants in four of them. In relation to the recent report of Kummerle *et al. *[16] the present study analyzes in more detail the actual impact of canakinmab on disease activity according to the disease phenotype. Globally, patients with a mild-intermediate MWS phenotype display complete control of disease activity maintaining the initial dosage of 2 mg/kg (or 150 mg) every 8 weeks, independent of their age. The majority of our CINCA patients required an increase in the dose to 4 mg/kg (or 300 mg) during the ACZ885D2306 trial. For most of them this dose adjustment was not sufficient to achieve complete control of disease activity, and a progressive increase of the dosing frequency was needed in the following 12 months. In the majority of the patients the main reason for interval adjustment was the presence of a persistent elevation of acute phase reactants, despite complete control of the clinical manifestations. In one patient with severe CINCA (patient number 2), the clinical manifestations persisted along with the elevation of acute phase reactants, despite the previous increase of the dose to 300 mg every 6 weeks. In this case parents did not allow further increase in the frequency of the administration or escalation of the dose and asked for a return to the anakinra regimen.

The variability in the response to IL-1 blockers has been already observed in previous studies. The wide use of anakinra in all CAPS phenotypes has already provided evidence that patients with CINCA generally need higher doses to completely control disease activity compared to patients with a milder CAPS phenotype [11-13]. This is due to different reasons: first, the impact of different *NLRP3 *mutations on the levels of secretion of IL-1β from monocytes is variable, with higher levels detected in the severe CINCA phenotype [7,18]. Second, this phenotype is characterized by the inflammatory involvement of the CNS and inner ear. In non-human primates, the diffusion of anakinra in the CNS is proportional to the systemic dose [19], supporting the need for an increasing dose to achieve complete control of CNS manifestations [13]. Third, most of the patients with CINCA are treated during childhood. Even if there are no clear data on the pharmacokinetic of IL-1 blockers in humans so far, it is conceivable that the bioavailability of these drugs in children is lower than in adults.

Interestingly in our study, all patients who did not achieve complete control of acute phase reactants at the last follow-up of treatment with canakinumab, displayed the same pattern at the moment of the anakinra withdrawal, supporting the need to identify the proper dose of IL-1 blockers on an individual basis. This issue might be due to the different impact of the *NLRP3 *mutations on disease severity. This seems to be the case for the T348M mutation that in our experience determined an increased dosage regimen independently from our classification according to the disease phenotype. On the other hand the need to change the treatment schedule was also observed in CINCA patients who were negative for germ-line *NLRP3 *mutations.

In any case, all partial responders displayed a CINCA phenotype, or had high body weight. In this line, the existence of a single formulation for both anakinra (100 mg) and canakinumab (150 mg) represents a clear limitation for the proper management of the more severely affected patients.

In the present study we also compared the response to treatment in CAPS patients after 12 months of canakinumab treatment with those registered at the moment of anakinra withdrawal. This analysis has a number of limitations related to the retrospective collection of data and to the temporal gap (mean 23 months, range 18 to 36) between the last administration of anakinra and the evaluation of the patients after 12 months of treatment with canakinumab. Despite these limitations, our preliminary observations support the clinical perception of substantial equivalence of the two IL-1 blockers in the control of disease manifestations and their role in providing a sustained improvement in HRQoL [20]. With no doubt, the possibility of avoiding daily injections represented the main reason for observed better performance according to scores for the psychological items, when patients are treated with canakinumab. Due to its higher molecular weight, canakinumab has a much lower probability of reaching the CNS compared to anakinra. Despite the theoretical concern about the minor efficacy of canakinumab for CNS manifestations, none of the CINCA patients enrolled in the study had evidence of deterioration in CNS involvement during canakinumab treatment. However, in the long run further follow-up in larger series is needed to clarify this relevant issue.

So far, the safety profile reported for anakinra in the long run is rather good [11-13]. In our recent experience local erythematosus skin reaction at the injection site (28.5% of treated patients), excessive weight gain (14.2%) and severe oral aphthosis (7.1%) were the most relevant reported adverse events [11]. In this study canakinumab confirmed optimal tolerability together with a good safety profile [15,16]. Infections should be carefully and promptly recognized for proper and aggressive treatment. In the case of infectious cellulitis the previous use of canakinumab did not interfere with the time of resolution of this complication. To our knowledge this clinical manifestation has never been described in association with CAPS, or as a possible adverse event during treatment with biologic agents.

Our study shows that the CAPS phenotype, rather than patient age, represents the main variable to determine the need of more frequent administration of the drug at higher doses. Due to the age limit of 4 years in the ACZ885D2306 study, no information on very young children is available so far. Taking into account these limitations, we suggest that it is reasonable for pediatric patients above the age of 4 years who have MWS to be treated with the proposed schedule of 2 mg/kg every 8 weeks. However, patients with the severe CINCA phenotype should be more aggressively treated from the beginning. In our opinion monthly administration of 4 mg/kg should be the starting dose, with a possible progressive weekly decrease of the dosing frequency according to the clinical and laboratory response. Notably, this schedule has been recently proposed for systemic onset juvenile arthritis (SoJIA), so far with a satisfactory safety profile [21].

Further studies of both anakinra and canakinumab in larger pediatric serial studies are needed, including careful analysis of the pharmacokinetics in children younger than 4 years of age and long-term follow-up, in order to achieve a rational approach with IL-1 blockers for the broad phenotypic spectrum of CAPS in both children and adults.

## Conclusions

The use of canakinumab in daily practice is associated with persistent satisfactory control of disease activity but needs progressive dose adjustments in more severely affected patients. In this respect, the clinical phenotype, rather than the patient's age, represents the main variable that determines the indication to start treatment with more frequent administration of the drug at higher doses in patients with CINCA.

## Abbreviations

CAPS: cryopyrin associated periodic syndromes; CHQ-PF 50: 50-item version of the Child Health Questionnaire; CINCA: chronic infantile neurological cutaneous and articular syndrome; CNS: central nervous system; CRP: C-reactive protein; FCAS: familial cold autoinflammatory syndrome; HRQoL: health-related quality of life; IL: interleukin; JIA: juvenile arthritis; MWS: Muckle-Wells syndrome; SAA: serum amyloid A protein.

## Competing interests

F Zulian has received consultancy fees from Novartis. A Martini has received payment from Novartis for service on speakers' bureaus. M Gattorno has received consultancy fees and honoraria Novartis and SOBI Biovitrum for meeting presentations. R Caorsi, L Lepore, M Alessio, A Stabile, A Insalaco, M Finetti, A Battagliese, G Martini and C Bibalo have no competing interests to declare.

## Authors' contributions

RC collected and analyzed the data and helped to draft the manuscript. FZ, LL, MA, AS, and AI contributed to the design and analysis of the study. MF, AB, GM, and CB collected the data. AM contributed to the design of the study and helped to draft the manuscript. MG conceived and coordinated the study and wrote the manuscript. All authors read and approved the final manuscript.
